# Sentrin‐specific protease 3 (SENP3)-mediated Krüppel-like factor 4 (KLF4) deSUMOylation regulates vascular smooth muscle cell phenotypic switching in atherosclerosis

**DOI:** 10.1186/s43556-025-00365-5

**Published:** 2025-11-27

**Authors:** Zi Wang, Yinan Wang, Ruosen Yuan, Qingqi Ji, Yanjie Li, Huanhuan Huo, Guo Zhou, Xiangming Yan, Linghong Shen, Zhaohua Cai, Ben He

**Affiliations:** 1 Department of Cardiology, Ren Ji Hospital, Shanghai Jiao Tong University School of Medicine, Shanghai, 200127 China; 2 Department of Cardiology, Shanghai Chest Hospital, Shanghai Jiao Tong University School of Medicine, Shanghai, 200030 China; 3 State Key Laboratory of Systems Medicine for Cancer, Renji-Med X Clinical Stem Cell Research Center, Ren Ji Hospital, Shanghai Jiao Tong University School of Medicine, Shanghai, 200127 China; 4 Department of Cardiology, Rui Jin Hospital, Shanghai Jiao Tong University School of Medicine, Shanghai, 200025 China

**Keywords:** SENP3, KLF4, SUMOylation, Vascular smooth muscle cells, Phenotypic switching, Atherosclerosis

## Abstract

Phenotypic switching of vascular smooth muscle cells (VSMCs) from a contractile toward a synthetic phenotype plays a critical role in atherosclerosis. Although the redox-sensitive sentrin/Small Ubiquitin-like Modifier (SUMO)-specific protease 3 (SENP3), which preferentially deconjugates SUMO2/3, has been linked to oxidative stress, its role in atherosclerosis remains poorly defined. In this study, we demonstrate that SENP3 is significantly upregulated in human and mouse atherosclerotic lesions and in VSMCs exposed to pro-atherogenic stimuli. Using smooth muscle-specific *Senp3* knockout mice (*ApoE*^−/−^;*Senp3*^*flox/flox*^;*Tagln-Cre)* and SENP3-knockdown VSMC models, we show that SENP3 deficiency preserves the contractile phenotype of VSMCs, suppresses their proliferation and migration, and attenuates atherosclerotic lesion development. Specifically, *Senp3* deletion reduces plaque formation and lipid accumulation, while enhancing collagen deposition and fibrous cap stability, shifting plaques toward a more stable phenotype. Mechanistically, we determined the transcription factor Krüppel-like factor 4 (KLF4) as a direct substrate of SENP3. SENP3 deSUMOylates KLF4 at lysine 278, thereby inhibiting its ubiquitin-mediated degradation and increasing its stability. We further show that SUMOylation at K278 serves as an intrinsic brake on KLF4-mediated phenotypic switching in VSMCs. These findings reveal that SENP3‐driven deSUMOylation of KLF4 regulates VSMC phenotypic switching in atherosclerosis, highlighting the SENP3/KLF4 axis as a pivotal regulator of vascular plasticity and a promising therapeutic target for atherosclerotic disease.

**Supplementary Information:**

The online version contains supplementary material available at 10.1186/s43556-025-00365-5.

## Introduction

Atherosclerotic cardiovascular disease (ASCVD) remains the primary driver of global mortality and disability, presenting a substantial public health challenge [1, 2]. According to the 2019 Global Burden of Disease Study, ASCVD was responsible for nearly 17.9 million deaths worldwide, with ischemic heart disease and cerebrovascular disease representing the two largest contributors to mortality and years of life lost [3, 4]. This immense clinical and socioeconomic burden underscores a pressing imperative to elucidate the molecular mechanisms of atherosclerosis and discover novel therapeutic targets.

Atherosclerosis is widely recognized as a chronic inflammatory disorder characterized by the dysfunction and pathological activation of multiple vascular cell types, including endothelial cells, immune cells, and vascular smooth muscle cells (VSMCs) [5, 6]. VSMCs, in particularly, exhibit remarkable phenotypic plasticity and play a central role throughout all stage of atherosclerotic plaque development –from initiation and progression to ultimate rupture [7, 8]. The phenotypic switching of VSMCs is a pivotal event in this process, characterized by a shift from a contractile, quiescent state to a synthetic phenotype that promotes proliferation and migration [9, 10]. This transition is marked by the downregulation of key contractile markers, including α-smooth muscle actin (αSMA), smooth muscle myosin heavy chain (SM-MHC), and smooth muscle 22α (SM22α), accompanied by increased proliferation, migration, and extracellular matrix production [11, 12]. Despite the established importance of VSMC plasticity, the precise molecular mechanisms that regulate this process within atherosclerotic lesions remain incompletely understood.

Post-translational modifications (PTMs) constitute an essential regulatory mechanism in vascular biology, enabling dynamic control of protein function in response to pathological stimuli [13]. Among these, SUMOylation, a reversible PTMs where Small Ubiquitin-like Modifier (SUMO) proteins are covalently linked to lysine residues on target proteins, serves as a critical regulator of numerous cellular processes such as protein stability, subcellular localization, and transcriptional activity [14]. DeSUMOylation, catalyzed by a family of sertrin/SUMO-specific proteases (SENPs), reverses this modification, and finely regulates substrate function. The SENP family includes six known members (SENP1–3 and 5–7) [15], among which SENP3 is distinguished by its redox sensitivity and pronounced preference for cleaving SUMO2/3 conjugates [16]. Although SENP3 has been implicated in cancer and metabolic disease through its deSUMOylation of substrates such as β-catenin, YAP1 and CTH [17–22], its potential role in atherosclerosis and VSMC phenotypic modulation has not been examined.

Krüppel-like factor 4 (KLF4) is a zinc-finger transcription factor implicated in the control of cellular differentiation, proliferation, and inflammation responses [23–25]. In the vascular system, KLF4 has been established as a central mediator of VSMC plasticity; its genetic deletion in smooth muscle cells significantly attenuates atherosclerosis and aortic aneurysm formation in murine models [26–28]. The regulation of KLF4 is achieved through multiple mechanisms, spanning transcriptional control and various PTMs, for instance, phosphorylation, acetylation, ubiquitination and SUMOylation [29, 30]. Previous studies have demonstrated that SUMOylation modulates KLF4 activity—for instance, SUMO1 conjugation promotes KLF4-mediated M2 macrophage polarization, while SENP1-mediated de-SUMOylation of KLF4 drives M1 polarization via NF-κB activation and influences cancer cell behavior [31]; Nie et al. demonstrated that SUMO1 conjugation of KLF4 is critical for transcriptional corepressor binding at the p21 promoter, a key event in modulating VSMC proliferation [32]. However, the potential regulation of KLF4 by SENP3, and functional consequences of such regulation in VSMCs and atherosclerosis, have not been explored.

Based on the established roles of SENP3 in regulating key signaling molecules and the critical function of KLF4 in VSMC biology, we hypothesize that SENP3 promotes VSMCs phenotypic switching and atherosclerosis progression through deSUMOylation and stabilizing KLF4. Here, we show that SENP3 expression is upregulated in human and mouse atherosclerotic lesions as well as in VSMCs exposed to pro-atherogenic stimuli. Using smooth muscle-specific *Senp3* knockout mice on an *ApoE*^*−/−*^ background, we demonstrated that *Senp3* deletion attenuates atherosclerosis, promotes plaque stability, and preserves the contractile VSMCs phenotype. Mechanistically, we identify KLF4 as a direct substrate of SENP3 and pinpoint lysine 278 as the critical residue required for SENP3-mediated deSUMOylation of KLF4. Our findings define the SENP3-KLF4 axis as a novel regulatory mechanism controlling VSMC plasticity and atherosclerotic progression, underscoring its promise as a therapeutic target for cardiovascular disease.

## Results

### Atherogenic stimuli upregulate SENP3 expression in VSMCs in vitro and in vivo

**Fig. 1:**
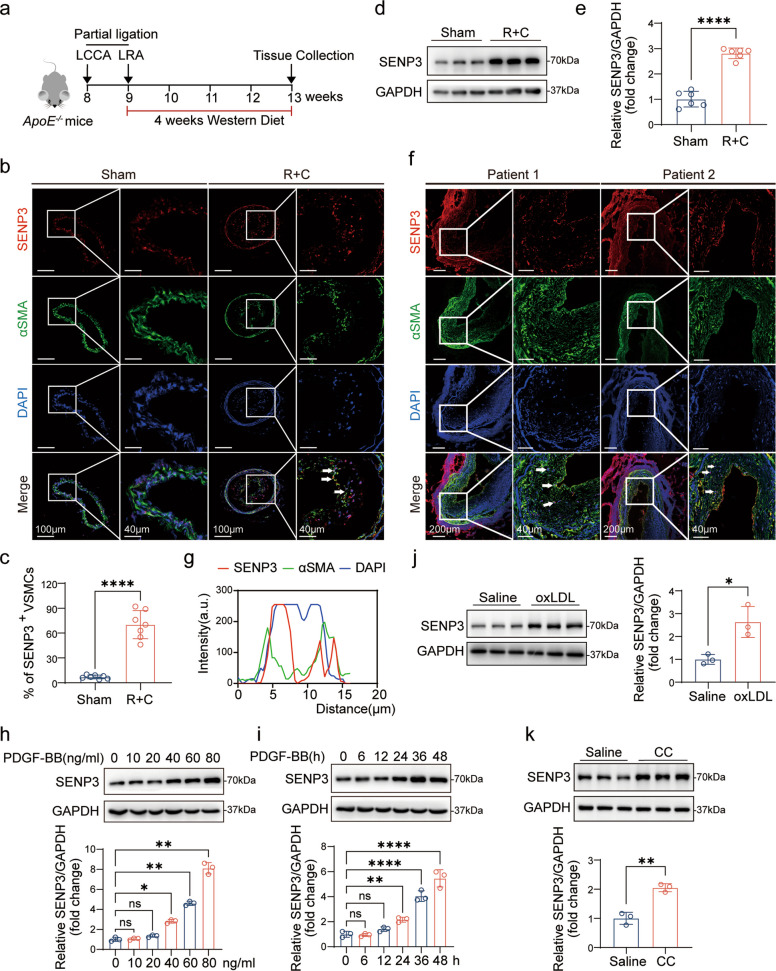
SENP3 is elevated in atherosclerotic lesions and induced by atherogenic stimuli. **a** Schematic diagram of the experimental timeline for the mouse atherosclerosis model. **b** Representative immunofluorescence images of SENP3 (red) and α-SMA (green) in carotid artery sections. Scale bar: 100 μm. **c** Quantification of SENP3-positive VSMCs in carotid arteries of *ApoE*^*⁻/⁻*^ mice subjected to ligation and sham surgery (*n* = 7). **d** Western blot analysis of SENP3 protein expression in aortic lysates from *ApoE*.^*⁻/⁻*^ mice subjected to ligation and sham surgery. **e** Quantification of SENP3 protein levels normalized to GAPDH from (**d**) (*n* = 6). **f** Representative immunofluorescence images of SENP3 (red) and α-SMA (green) in human atherosclerotic popliteal arteries. Scale bar: 200 μm. **g** Quantification of nuclear SENP3 and DAPI colocalization in human samples. **h, i** Western blot analysis of SENP3 expression in primary rat VSMCs treated with PDGF-BB at increasing concentrations (**h**) or for varying durations (**i**) (*n* = 3). **j, k** SENP3 protein levels in primary rat VSMCs after 24 h treatment with oxidized LDL (oxLDL, 20 μg/mL) (**j**) or cholesterol crystals (CC, 1 mg/mL) (**k**) (*n* = 3). Data are presented as mean ± SEM; statistical significance was assessed by one-way ANOVA or unpaired Student’s t-test. *p < 0.05; **p < 0.01; ***p < 0.001; ****p < 0.0001

To investigate the role of SENP3 in atherosclerosis, we first assessed its expression pattern under pro-atherogenic conditions. We employed an established accelerated atherosclerosis model involving partial ligation of the left renal artery (LRA) and the left common (LCCA), combined with Western diet feeding, in *ApoE*^*−/−*^ mice [33, 34] (Fig. 1a). Immunofluorescence analysis of carotid artery sections revealed a marked upregulation of SENP3 within atherosclerotic lesions. Notably, SENP3 was predominantly localized to αSMA-positive VSMCs in plaque areas (Fig. 1b), with quantitative analysis confirming a significant increase in SENP3 expression (Fig. 1c). To further quantify SENP3 protein levels in vivo, we performed Western blot analysis on aortic tissues isolated from *ApoE*^*⁻/⁻*^ mice that had undergone partial ligation or sham surgery. Consistent with the immunofluorescence data, SENP3 level was markedly elevated in the ligation group relative to the sham controls (Fig. 1d). Densitometric analysis further confirmed this increase (Fig. 1e).

To evaluate the clinical relevance of these findings, we examined human popliteal arteries with advanced atherosclerotic lesions. Consistent with the murine data, SENP3 expression was significantly elevated and primarily nuclear within VSMCs of human lesions (Fig. 1f), as supported by colocalization analysis (Fig. 1g).

We further characterize SENP3 regulation using an in vitro model of VSMC phenotypic modulation. SENP3 expression was upregulated in a dose- and time-dependent manner in primary rat VSMCs following exposure to atherogenic stimuli—namely, platelet-derived growth factor-BB (PDGF-BB), oxidized low-density lipoprotein (oxLDL), or cholesterol crystals (Fig. 1h-i; Fig. S1a-b). Specifically, PDGF-BB increased SENP3 protein levels across multiple concentrations and time points, while oxLDL (10 μg/mL) and cholesterol crystals (1 mg/mL) significantly elevated SENP3 after 24 h (Fig. 1j-k).

Together, these findings demonstrated that SENP3 is consistently upregulated in VSMCs under atherogenic conditions both animal models and cell culture systems, suggesting a potential role in VSMC phenotypic switching during atherosclerosis.

### Smooth muscle cell (SMC)-specific deletion of SENP3 attenuates atherosclerosis and enhances plaque stability

**Fig. 2:**
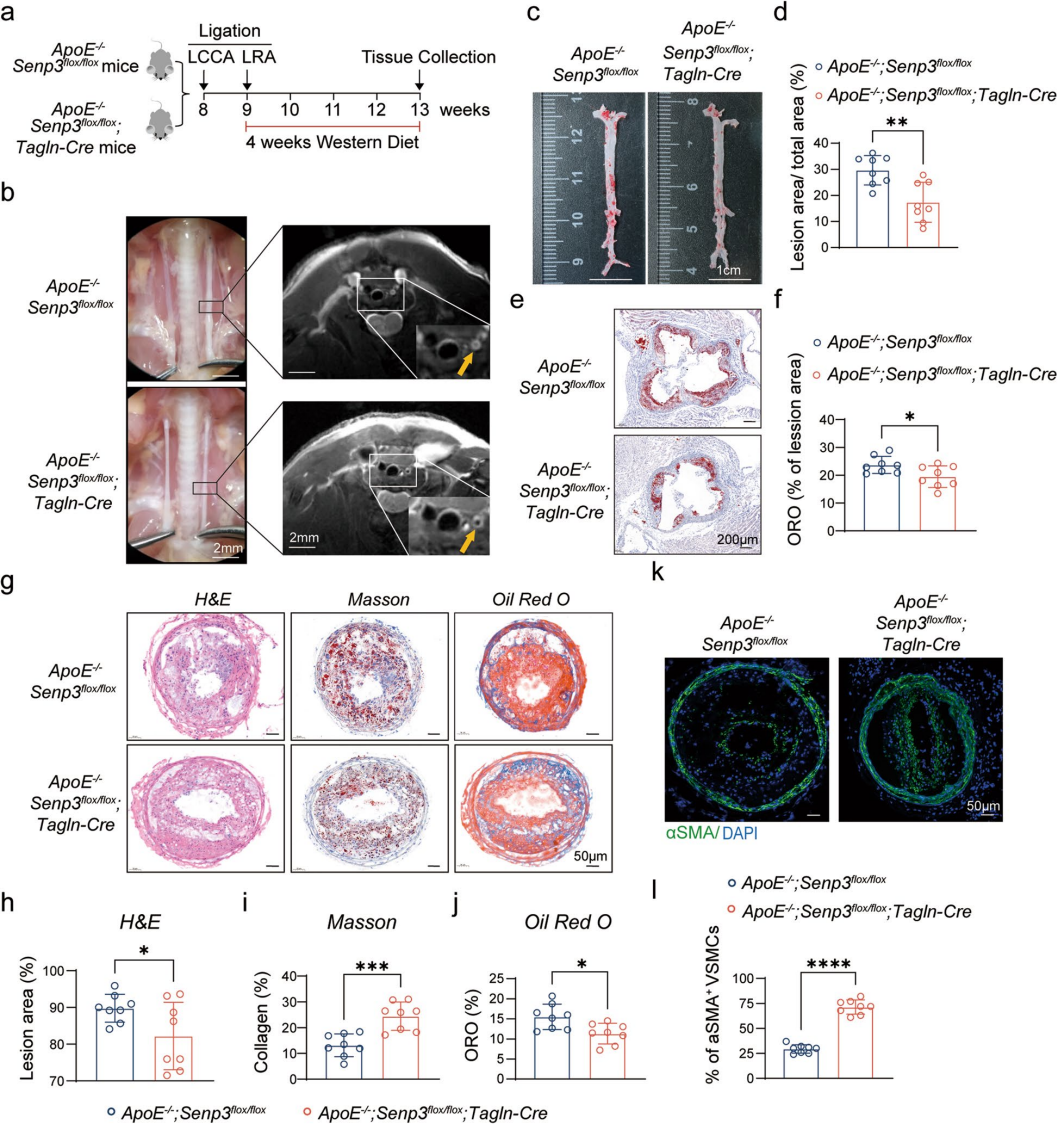
SMC-specific deletion of SENP3 reduces atherosclerotic burden and improves plaque stability. **a** Experimental timeline of partial ligation model and Western diet feeding. **b** Representative magnetic resonance (MR) images showing carotid artery plaque size in *ApoE*^−/−^;*Senp3*^*flox/flox*^ and *ApoE*^−/−^;*Senp3*.^*flox/flox*^;*Tagln-Cre* mice. Scale bar: 2 mm. **c** Representative en face Oil Red O-staining of aortas. Scale bar: 1 cm. **d** Quantification of total atherosclerotic lesion area (*n* = 8). **e** Representative Oil Red O staining of aortic root sections. Scale bar: 200 μm. **f** Quantification of lipid area in aortic root lesions (*n* = 8). **g** Representative hematoxylin and eosin (H&E), Masson’s trichrome, and Oil Red O staining of LCCA sections. Scale bar: 50 μm. **h** Quantification of lesion area in LCCA sections (*n* = 8). **i** Quantification of collagen content (%) in plaques (*n* = 8). **j** Quantification of lipid deposition intensity in LCCA sections (*n* = 8). **k** Representative immunofluorescence images of α-SMA (green) and DAPI (blue) staining in LCCA plaques. Scale bar: 50 μm. **l** Quantification of α-SMA-positive cells per field within fibrous caps (*n* = 8). Data are presented as mean ± SEM. Statistical significance was determined by unpaired Student’s t-test. *p < 0.05; **p < 0.01; ***p < 0.001; ****p < 0.0001

To define the role of SENP3 in atherosclerosis, we generated *Senp3*^*flox/flox*^;*Tagln-Cre* mice on an *ApoE*^−/−^ background, enabling SMC-specific *Senp3* knockout [35] (Fig. S2). Following partial ligation of the LRA and LCCA, mice were fed a Western diet for 4 weeks (Fig. 2a). Non-invasive magnetic resonance imaging (MRI) revealed a substantial reduction in carotid plaque size in *ApoE*^−/−^;*Senp3*^*flox/flox*^;*Tagln-Cre* mice compared to controls (Fig. 2b). En face Oil Red O staining of the entire aorta showed a significant decrease in total atherosclerotic lesions area (Fig. 2c-d), and cross-sections of the aortic root displayed reduced lipid accumulation in *Senp3*-deficient mice (Fig. 2e-f). Histological evaluation of LCCA sections further demonstrated that *Senp3* deletion resulted in smaller lesion size by hematoxylin and eosin (H&E) staining (Fig. 2g-h), increased collagen content by Masson’s trichrome staining (Fig. 2g, i), and reduced lipid deposition by Oil Red O staining (Fig. 2g, j). Immunofluorescence analysis also indicated a higher density of αSMA^+^ VSMC within the fibrous cap of *Senp3*-deficient mice (Fig. 2k-l), suggesting enhanced plaque stability.

These results indicate that SMC-specific deletion of *Senp3* reduces atherosclerotic plaque formation and promotes a stable plaque phenotype.

### SENP3 deficiency attenuates phenotypic switching and inflammatory responses in VSMCs

**Fig. 3:**
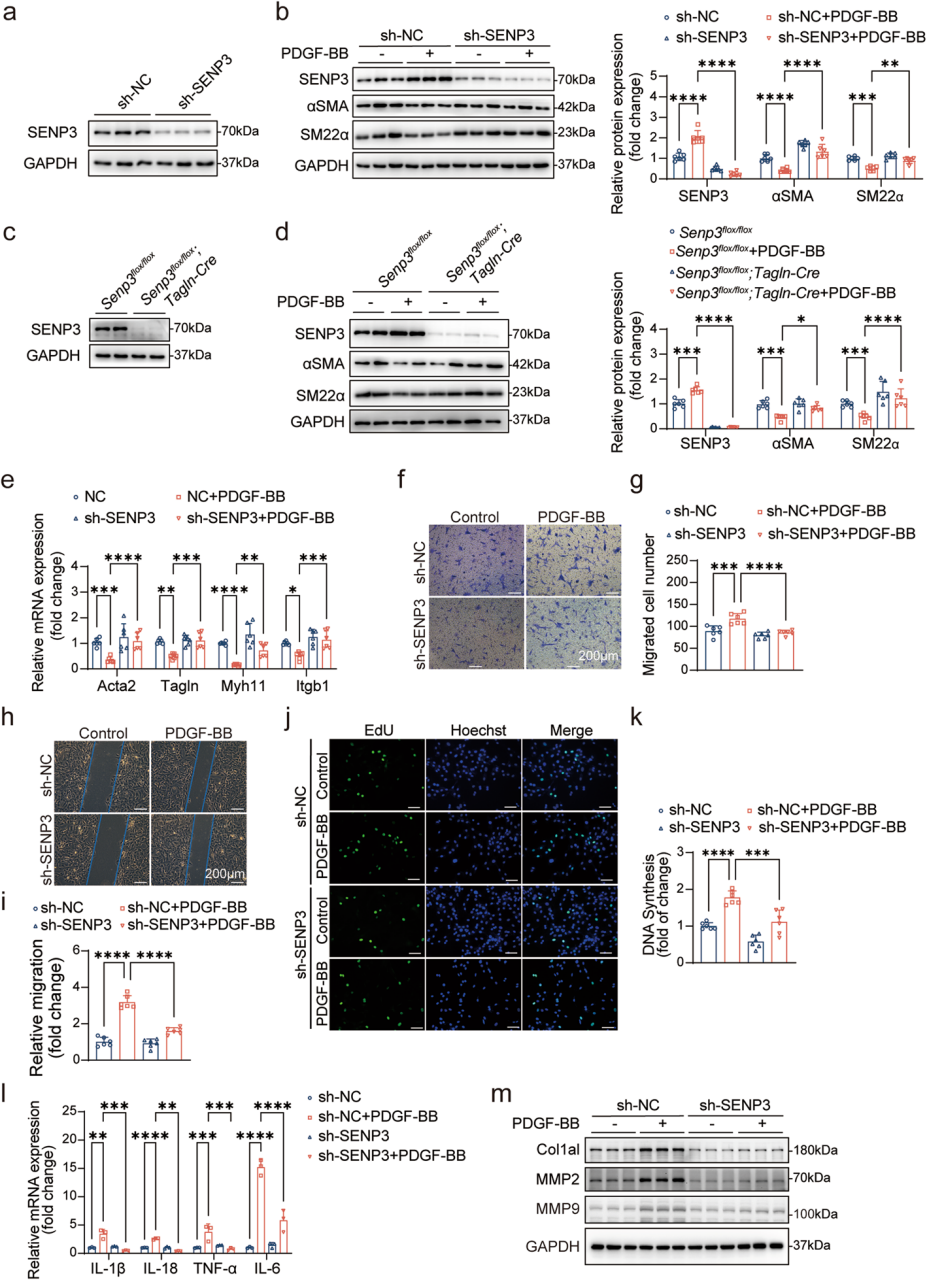
SENP3 knockdown suppresses phenotypic switching, migration, proliferation, and inflammation in VSMCs. **a** Western blot analysis of SENP3 protein expression in primary rat VSMCs transduced with lentiviral particles carrying sh-scramble (sh-NC) or sh-SENP3 (*n* = 3). **b** Protein levels of SENP3, α-SMA, and SM22α in VSMCs infected with sh-NC or sh-SENP3 lentivirus and treated with PDGF-BB (40 ng/ml) for 24 h (*n* = 6). **c** SENP3 protein expression in primary VSMCs isolated from *Senp3*^*flox/flox*^ and *Senp3*^*flox/flox*^;*Tagln-Cre* mice. **d** Protein levels of SENP3, α-SMA, and SM22α in VSMCs from *Senp3*^*flox/flox*^ and *Senp3*.^*flox/flox*^; *Tagln-Cre* mice treated with PDGF-BB (40 ng/ml) for 24 h (*n* = 6). **e** mRNA expression of contractile marker genes *Acta2, Tagln, Myh11,* and *Itgb1* in rat VSMCs infected with sh-NC or sh-SENP3 lentivirus and stimulated with PDGF-BB (40 ng/mL) for 24 h (*n* = 6). **f, g** Transwell migration assays images and quantification of migrated cells per field in VSMCs infected with sh-NC or sh-SENP3 lentivirus and stimulated with PDGF-BB (*n* = 6). Scale bar: 200 μm. **h, i** Scratch-wound assays images and quantification of relative cell migration (fold change) in VSMCs infected with sh-NC or sh-SENP3 lentivirus and treated with PDGF-BB (*n* = 6). Scale bar: 200 μm. **j, k** EdU incorporation assays images and percentage of EdU-positive cells (fold change) in VSMCs infected with sh-NC or sh-SENP3 lentivirus and stimulated with PDGF-BB (*n* = 6). Scale bar: 100 μm. **l** mRNA expression of IL-1β, IL-18, TNF-a, IL-6 in VSMCs under PDGF-BB stimulation (*n* = 3). **m** Western blot analysis of Col1a1, MMP2, and MMP9 protein levels in VSMCs under the indicated treatments (*n* = 3). Data are presented as mean ± SEM. Statistical significance was assessed by one-way ANOVA with multiple comparisons. *p < 0.05; **p < 0.01; ***p < 0.001; ****p < 0.0001

To explore how SENP3 modulates VSMC plasticity at the cellular level, we knocked down SENP3 in primary rat VSMCs using lentivirus-delivered shRNA (Fig. 3a). Under PDGF-BB stimulation, *SENP3* knockdown prevented the downregulation of contractile markers (αSMA and SM22α) (Fig. 3b). Consistent with this finding, primary VSMCs isolated from *Senp3*^*flox/flox*^*;Tagln-Cre* mice maintained higher expression of contractile proteins compared to controls after PDGF-BB treatment (Fig. 3c-d). qPCR analysis further confirmed that SENP3 silencing prevented PDGF-BB-induced suppression of contractile genes, including *Acta2*, *Tagln*, *Myh11*, and *Itgb1* (Fig. 3e).

Functional assays revealed SENP3 deficiency significantly impaired PDGF-BB-induced migration ability of VSMC, as evidenced by both Transwell and scratch wound assays (Fig. 3f-i). Furthermore, EdU incorporation assays revealed that SENP3 knockdown markedly reduced VSMCs proliferation under PDGF-BB stimulation (Fig. 3j-k). To further explore the inflammation aspect in phenotypic switching, we quantified the expression of relevant pro-inflammatory mediators. qPCR analysis indicated that SENP3 deficiency significantly suppressed the mRNA levels of key inflammatory mediator (IL-1β, IL-18, TNF-α, and IL-6) in response to PDGF-BB stimulation (Fig. 3l).

We further investigated the effect on matrix metalloproteinases (MMPs), given their pivotal role in extracellular matrix remodeling and plaque vulnerability. Western blot analysis indicated that SENP3 knockdown suppressed the PDGF-BB-induced upregulation of MMP2, MMP9, and Col1a1 (Fig. 3m), implicating a role for SENP3 in the regulation of VSMC-driven inflammation and plaque destabilization.

Together, these data indicate that SENP3 is essential for PDGF-BB-induced phenotype switching in VSMCs. Loss of SENP3 preserves the contractile phenotype while attenuating pro-inflammatory and synthetic responses.

### SENP3 overexpression promotes the acquisition of a synthetic phenotype in VSMCs

**Fig. 4:**
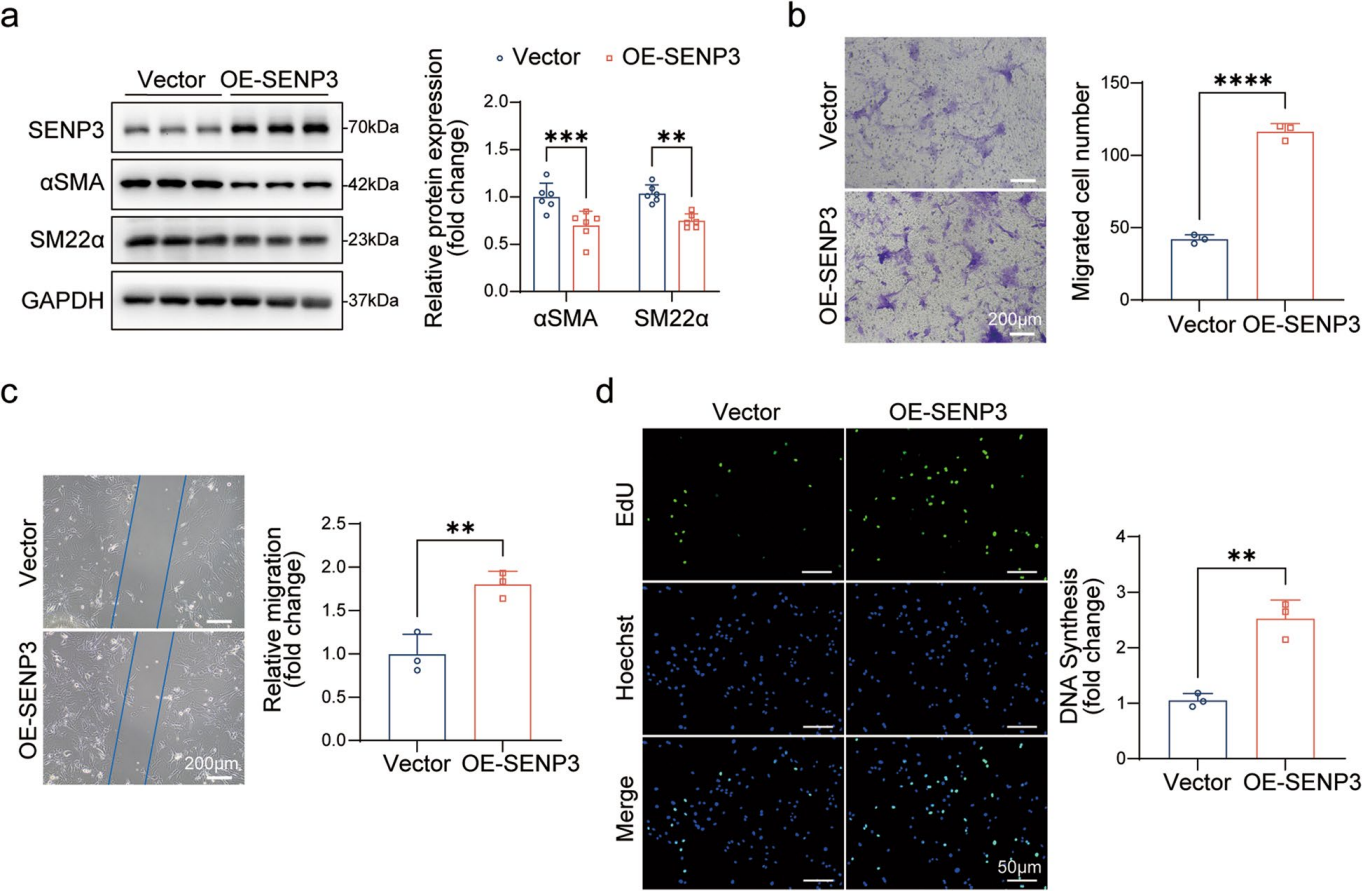
SENP3 overexpression drives synthetic phenotype. **a** Western blot analysis of αSMA and SM22αlevels in primary rat VSMCs transfected with empty vector (EV) or SENP3 overexpression plasmid (OE-SENP3) (*n* = 6). **b** Transwell migration assay images and quantification of migrated cells per field in EV- or OE-SENP3-transfected VSMCs (*n* = 3). Scale bar: 200 μm. **c** Scratch wound assay images and quantification of relative cell migration (fold change) in EV- or OE-SENP3-transfected VSMCs (*n* = 3). Scale bar: 200 μm. **d** EdU incorporation assay images and percentage of EdU-positive cells (fold change) in EV- or OE-SENP3-transfected VSMCs (*n* = 3). Scale bar: 200 μm. Data are presented as mean ± SEM. Statistical significance was determined by unpaired Student's t-test. *p < 0.05; **p < 0.01; ***p < 0.001; ****p < 0.0001

We next examined whether SENP3 overexpression is sufficient to drive phenotypic switching in VSMCs. Ectopic expression of SENP3 in primary rat VSMCs significantly downregulated the contractile markers αSMA and SM22α even in the absence of exogenous stimulation (Fig. 4a), indicating that SENP3 alone can suppress the contractile phenotype. Functionally, SENP3 overexpression enhanced VSMC migration, as shown by increased cell migration in both Transwell and scratch wound assays (Fig. 4b, c). Furthermore, SENP3 overexpression promoted cellular proliferation, as evidenced by elevated EdU incorporation (Fig. 4d).

These results indicate that SENP3 is sufficient to drive VSMC phenotypic switching toward a synthetic state, likely through its role in deconjugating SUMO2/3 from target proteins.

### SENP3 regulates VSMC phenotypic switching through KLF4

**Fig. 5:**
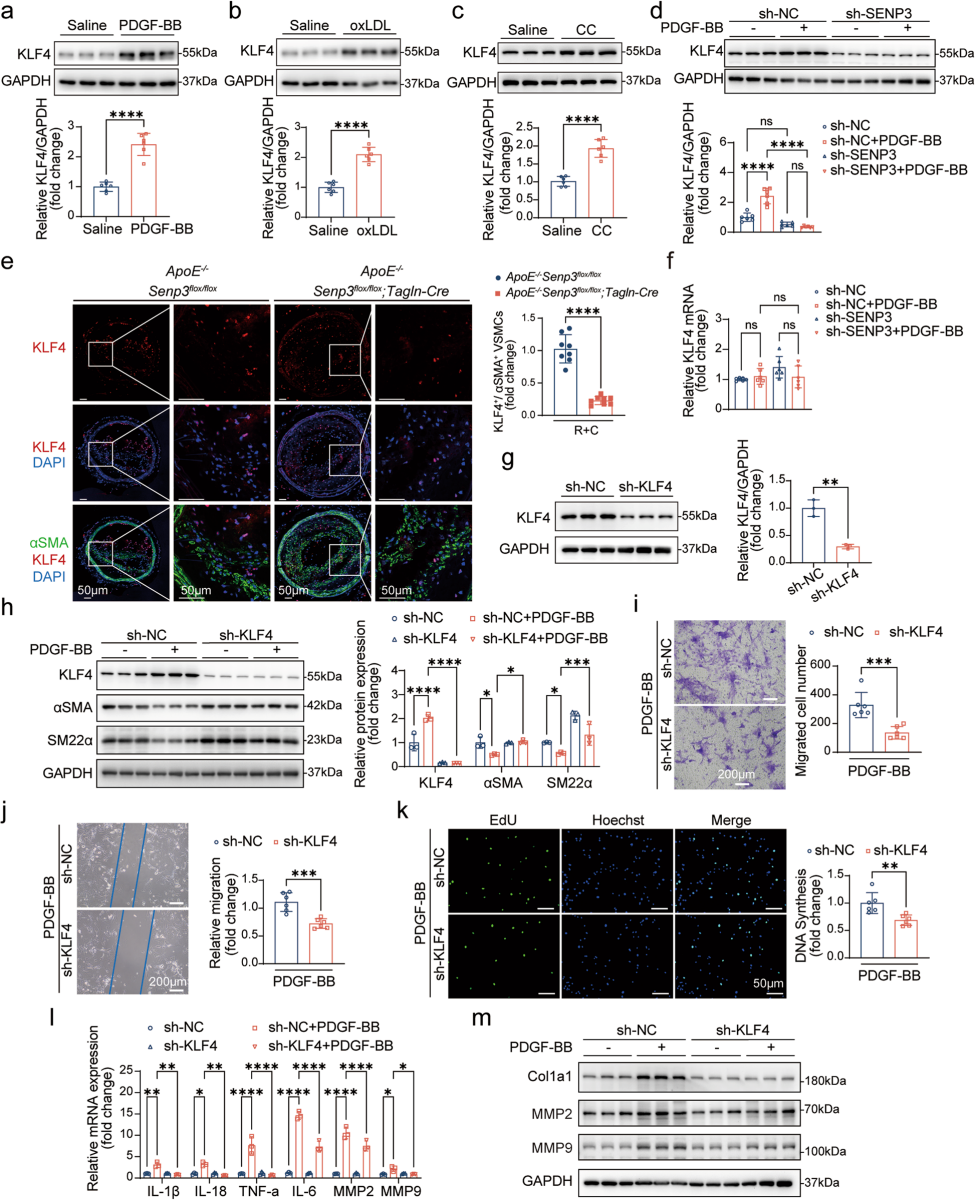
SENP3 regulates KLF4 expression in mediating pro-atherogenic responses in VSMCs. **a**-**c** Western blot analysis of KLF4 protein levels in primary rat VSMCs treated with PDGF-BB (40 ng/mL) (**a**), oxLDL (20 μg/mL) (**b**), or cholesterol crystals (CC, 1 mg/mL) (**c**) for 24 h (*n* = 6). **d** KLF4 protein levels in VSMCs infected with sh-NC or sh-SENP3 lentivirus and treated with PDGF-BB (40 ng/ml) for 24 h (*n* = 6). **e** Representative immunofluorescence images (left) of KLF4 (red) and α-SMA (green), and quantification (right) of KLF4^+^/α-SMA^+^ VSMCs in LCCA sections from *ApoE*^−/−^;*Senp3*^*flox/flox*^ and *ApoE*^−/−^;*Senp3*.^*flox/flox*^;*Tagln-Cre* mice (*n* = 8). Scale bar: 50 μm. **f** qPCR analysis of KLF4 mRNA expression in VSMCs treated with PDGF-BB (40 ng/ml) for 24 h (*n* = 6). **g** Validation of KLF4 knockdown efficiency in VSMCs infected with sh-NC or sh-KLF4 lentivirus (*n* = 3). **h.** Protein levels of α-SMA and SM22α in sh-NC or sh-KLF4 VSMCs treated with PDGF-BB (*n* = 3). **i** Transwell migration assays images and quantification of migrated cells per field in sh-NC or sh-KLF4 VSMCs treated with PDGF-BB (*n* = 6). Scale bar: 200 μm. **j** Scratch-wound assays images and quantification of relative cell migration (fold change) in sh-NC or sh-KLF4 VSMCs treated with PDGF-BB (*n* = 6). Scale bar: 200 μm. **k** Scratch-wound assays images and quantification of relative cell migration (fold change) in sh-NC or sh-KLF4 VSMCs treated with PDGF-BB (*n* = 6). Scale bar: 200 μm.** l** qPCR analysis of IL-1β, IL-18, TNF-α, and IL-6 mRNA in VSMCs under the same treatment condition (*n* = 3). **m** Western blot analysis of MMP2, MMP9, and Col1a1 protein levels in sh-NC or sh-KLF4 VSMCs treated with PDGF-BB (*n* = 3). Data are presented as mean ± SEM. Statistical significance was determined by one-way ANOVA or unpaired Student’s t-test. *p < 0.05; **p < 0.01; ***p < 0.001; ****p < 0.0001

To identify downstream effectors of SENP3, we investigated whether Krüppel-like factor 4 (KLF4)—a known central regulator of VSMC plasticity [36]—mediates the effects of SENP3. Western blot analysis revealed that treatment of primary rat VSMCs with PDGF-BB, oxLDL, or cholesterol crystals concurrently upregulated KLF4 protein levels (Fig. 5a-c). Notably, SENP3 knockdown prevented PDGF-BB-induced KLF4 upregulation (Fig. 5d). In vivo, immunofluorescence staining revealed reduced KLF4 levels alongside an increased density of αSMA^+^ VSMCs within plaques of *ApoE*^*−/−*^*;Senp3*^*flox/flox*^*;Tagln-Cre* mice compared to controls (Fig. 5e). KLF4 mRNA levels remained unchanged under PDGF-BB stimulation (Fig. 5f), suggesting that SENP3 regulates KLF4 at the post-transcriptional level.

We then directly tested whether KLF4 is required for phenotypic switching. Infection of primary rat VSMCs with lentiviral shRNA targeting KLF4 (sh-KLF4) efficiently reduced KLF4 protein expression (Fig. 5g). Notably, KLF4 knockdown markedly attenuated PDGF-BB-induced phenotypic switching, restoring the contractile markers expression (αSMA and SM22α) (Fig. 5h). Functionally, KLF4 deficiency suppressed PDGF-BB-promoted VSMC migration in Transwell and scratch wound assays (Fig. 5i-j) and inhibited cellular proliferation as measured by EdU incorporation (Fig. 5k).

Given the role of KLF4 in inflammation and matrix remodeling, we assessed its contribution to these processes. qPCR analysis demonstrated that KLF4 deficiency significantly reduced the mRNA expression of IL-1β, IL-18, TNF-α, and IL-6 (Fig. 5l). Consistently, the PDGF-BB-induced protein upregulation of MMP2, MMP9, and Col1a1 was inhibited by KLF4 knockdown, as evidenced by Western blot (Fig. 5m).

Together, these results support a model in which atherogenic stimuli upregulated SENP3, leading to KLF4-mediated promotion of synthetic phenotype switching in VSMCs. The SENP3-KLF4 axis thus represents a key regulatory mechanism of VSMC plasticity in atherosclerosis.

### SENP3 deSUMOylates and stabilizes KLF4 by targeting K278

**Fig. 6:**
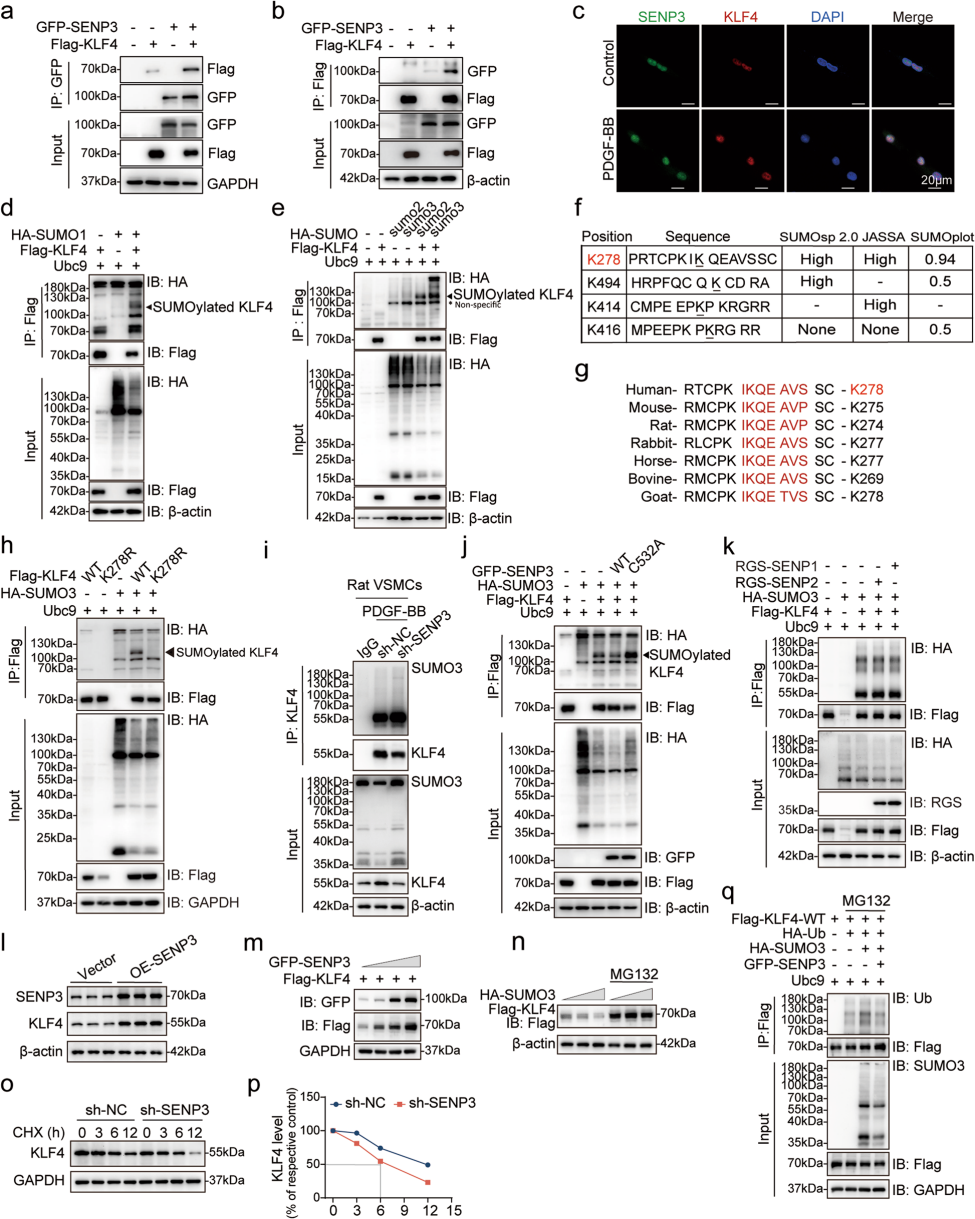
SENP3 deSUMOylates KLF4 at K278 and prevents its ubiquitin-mediated degradation. **a**, **b** Co-immunoprecipitation (co-IP) of exogenous interaction between SENP3 and KLF4 in HEK293T cells transfected with GFP-SENP3 and/or Flag-KLF4 plasmids for 36 h. **c** Representative confocal immunofluorescence images showing co-localization of SENP3 (green) and KLF4 (red) in primary rat VSMCs treated with PDGF-BB. Scale bar: 20 μm. **d**,** e** Assessment of KLF4 modification by SUMO1 (d) or SUMO2/3 (e) via IP and western blotting in HEK293T cells transfected with Flag-KLF4, HA-SUMO1/SUMO2/SUMO3, and Ubc9 for 36 h. **f** Prediction of SUMOylation sites in human KLF4 by bioinformatics tools. **g** Sequence alignment of the predicted SUMOylation sites of KLF4 across species, with conserved residues highlighted in red. **h** Analysis of SUMO3 modification on wide-type (WT) Flag-KLF4 or the K278R mutant in HEK293T cells expressing HA-SUMO3 and Ubc9. **i** Endogenous SUMO3-KLF4 conjugates in VSMCs transduced with sh-NC or sh-SENP3 and treated with PDGF-BB. **j** SUMO3-modified KLF4 levels in HEK293T cells expressing Flag-KLF4, HA-SUMO3, Ubc9, and either GFP-SENP3 WT or its catalytically inactive mutant (C532A). **k** Specificity of SENP3 versus SENP1 or SENP2 in regulating KLF4 SUMOylation, assessed by IP and western blot in HEK293T cells expressing Flag-KLF4, HA-SUMO3, Ubc9, and RGS-SENP1 or RGS-SENP2. **l** Endogenous KLF4 levels in primary rat VSMCs overexpressing empty vector (EV) or SENP3 expression. **m** Flag-KLF4 levels in HEK293T cells co-transfected with increasing doses of GFP-SENP3. **n** Flag-KLF4 levels in HEK293T cells transfected with Flag-KLF4 and increasing amounts of HA-SUMO3 for 36 h, in the presence or absence of MG132 (10 μM) for the last 10 h. **o**,** p** The half-life of endogenous KLF4 in primary rat VSMCs infected with NC lentivirus and shSENP3 lentivirus was examined by western blotting in the presence or absence of cycloheximide (CHX, 5 μg/mL) for the indicated time (*n* = 3). **q** KLF4 ubiquitination levels in HEK293T cells expressing HA-Ub, HA-SUMO3, and/or GFP-SENP3. Data are presented as mean ± SEM. Statistical significance was determined by unpaired Student’s t-test. *p < 0.05; **p < 0.01; ***p < 0.001; ****p < 0.0001

To determine if KLF4 serve as a direct substrate for SENP3, we initiated our mechanistic investigation. Co-immunoprecipitation assay confirmed a direct molecular interaction between SENP3 and KLF4 (Fig. 6a-b). Under PDGF-BB stimulation, confocal microscopy showed that SENP3 translocated from the nucleolus to the nucleoplasm and exhibited increased co-localization with KLF4 in cultured VSMCs (Fig. 6c), indicating a stimulus-dependent interaction within a functionally relevant compartment.

KLF4 underwent SUMO modification not only by SUMO1 (Fig. 6d), which is consistent with previous studies [31], but also robustly by SUMO2 and SUMO3 (Fig. 6e). Bioinformatics tools (SUMOsp 2.0, JASSA, and SUMOplot™) consistently predicted lysine 278 (K278) as a putative SUMOylation site which is highly conserved across species (Fig. 6 f-g). To validate this site, we generated plasmids encoding either wild-type or lysine-to-arginine (K-to-R) mutants of KLF4. Immunoprecipitation assays using anti-Flag M2 magnetic beads in HEK293T cells revealed that the K278R mutation abolished SUMO3 modification (indicated by arrowheads in Fig. 6h). SENP3 knockdown in primary rat VSMCs increased SUMO3-conjugated KLF4 levels under PDGF-BB stimulation (Fig. 6i). Conversely, overexpression of wild-type SENP3, but not its catalytically mutant SENP3-C532A, effectively reduced SUMO3-modified KLF4 (Fig. 6j). Furthermore, neither SENP1 nor SENP2 influenced KLF4 SUMOylation (Fig. 6k), underscoring the specificity of SENP3.

SENP3 overexpression led to a dose-dependently elevation of KLF4 protein levels in both primary VSMCs and HEK293T cells (Fig. 6l-m). In contrast, enhancing SUMO3 modification via SUMO3 overexpression reduced KLF4 abundance, an effect reversed by the proteasome inhibitor MG132 (Fig. 6n). To directly assess KLF4 turnover, we performed cycloheximide chase assays in VSMCs following SENP3 knockdown showed that SENP3 deficiency significantly shortened the half-life of KLF4 protein (Fig. 6o-p) without altering its mRNA levels (Fig. 5f), confirming that SENP3 regulates KLF4 post-translationally.

Given that KLF4 degradation is known to be governed by the ubiquitin–proteasome pathway [37], we examined whether SUMOylation influences KLF4 ubiquitination. SUMO3 overexpression enhanced KLF4 ubiquitination, which was attenuated by co-expression of SENP3 (Fig. 6q). These findings suggest that SENP3-mediated deSUMOylation counteracts SUMO3-induced ubiquitination and subsequent proteasomal degradation of KLF4.

Together, these results demonstrated that SENP3 directly binds and deSUMOylates KLF4 at K278, thereby inhibiting ubiquitin-mediated degradation and stabilizing KLF4 to promote atherogenic responses.

### SUMOylation at K278 attenuates KLF4-mediated phenotypic switching in VSMCs

**Fig. 7:**
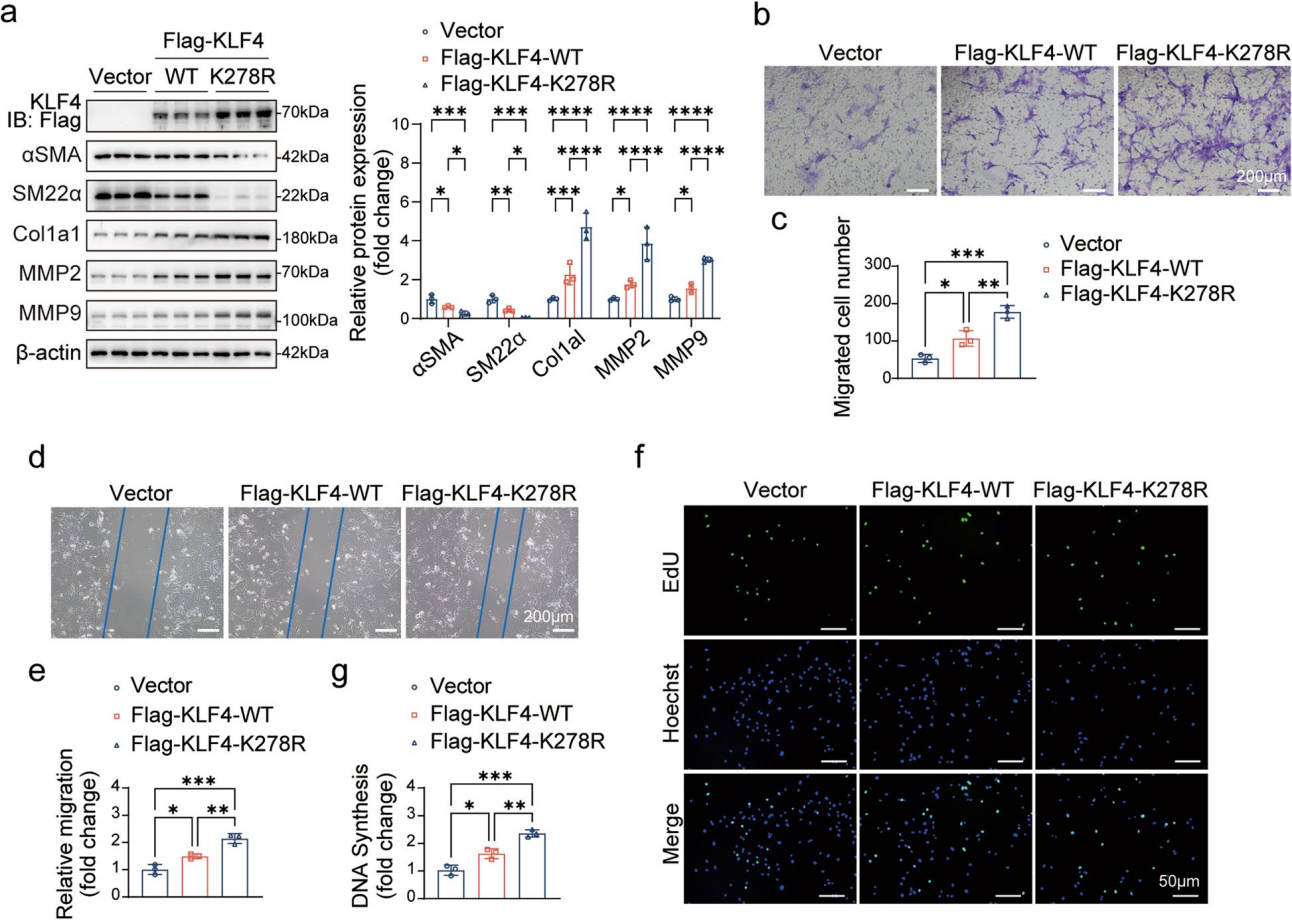
SUMOylation at K278 acts as an intrinsic brake on KLF4-mediated phenotypic switching. **a** Western blot analysis and quantification of key phenotypic markers (αSMA, SM22α, Col1a1, MMP2, MMP9) in primary rat VSMCs expressing wild-type KLF4 (KLF4-WT) or a SUMOylation-deficient mutant (KLF4-K278R) (*n* = 3). **b, c** Transwell migration assay images and quantification of migrated cells per field in VSMCs expressing KLF4-WT or KLF4-K278R (*n* = 3). **d, e** Scratch-wound assay images and quantification of relative cell migration (fold change) in VSMCs expressing KLF4-WT or KLF4-K278R (*n* = 3). **f, g** EdU incorporation assay images and percentage of EdU-positive cells (fold change) in VSMCs expressing KLF4-WT or KLF4-K278R (*n* = 3). Data are presented as mean ± SEM; statistical significance was determined by one-way ANOVA. *p < 0.05; **p < 0.01; ***p < 0.001; ****p < 0.0001

To determine the functional role of K278 SUMOylation in KLF4-driven phenotypic switching, we expressed wild-type KLF4 (KLF4-WT) or a SUMOylation-deficient mutant (KLF4-K278R) in primary rat VSMCs. Strikingly, expression of KLF4-K278R induced a more pronounced synthetic phenotype than KLF4-WT, as evidenced by a greater downregulation of contractile markers (αSMA, SM22α) and a stronger upregulation of synthetic mediators (Col1a1, MMP2 and MMP9) (Fig. 7a). Functionally, KLF4-K278R enhanced VSMC migration more potently than KLF4-WT in both Transwell and scratch-wound assays (Fig. 7b-e). Similarly, the K278R mutant promoted a stronger proliferative response compared to KLF4-WT (Fig. 7f-g).

These results demonstrated that SUMOylation at K278 serves as a critical intrinsic brake that constitutively limits the pro-atherogenic functions of KLF4. The enhanced synthetic phenotype driven by the SUMOylation-deficient mutant highlights the importance of SENP3-dependent deSUMOylation in fully activating KLF4-mediated phenotypic switching during atherosclerosis.

## Discussion

**Fig. 8:**
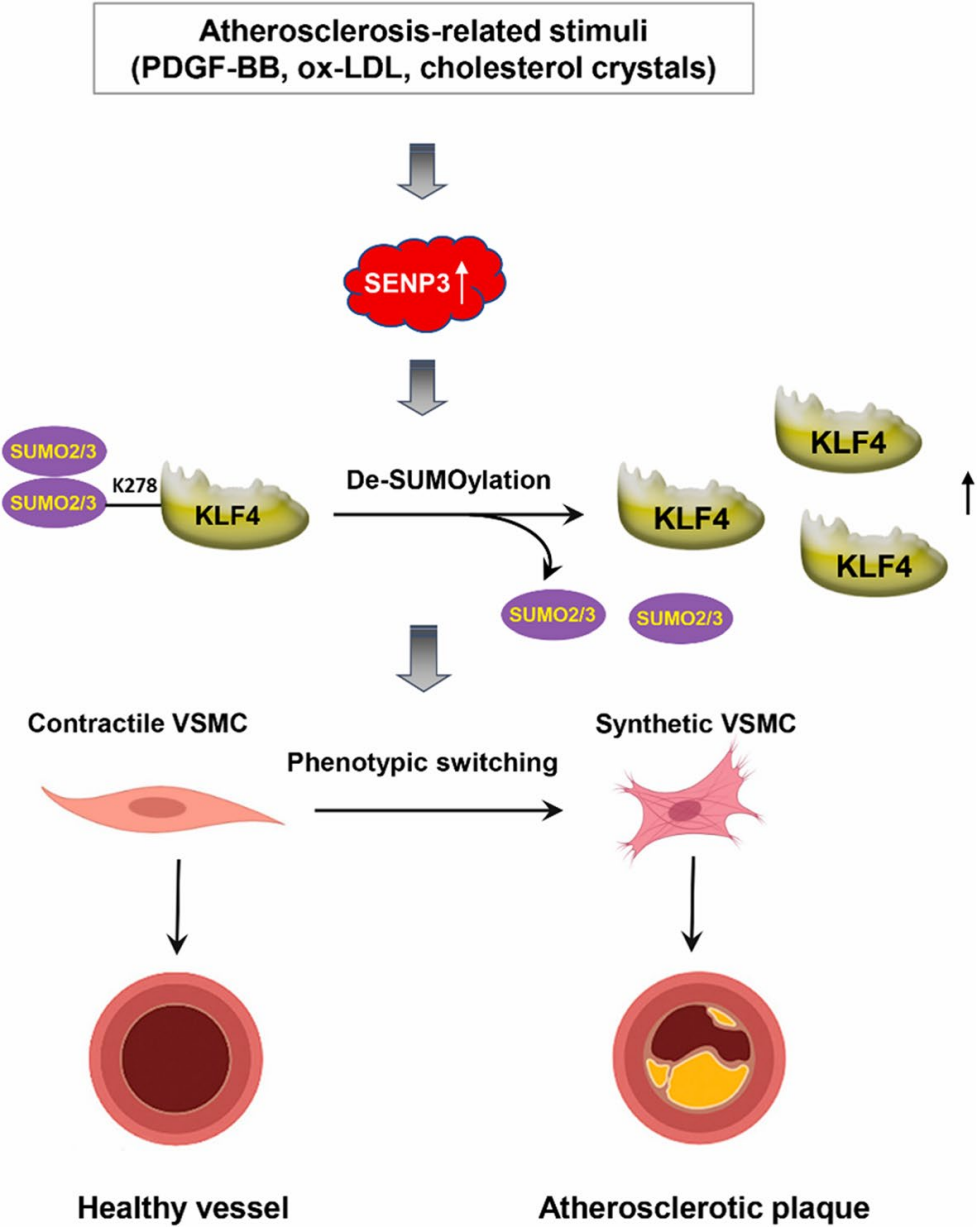
Schematic model of the SENP3-KLF4 axis in atherogenic VSMC phenotypic switching. Atherogenic stimuli induced SENP3 upregulation in VSMCs, where it catalyzes deSUMOylation of KLF4. This post-translational modification stabilizes KLF4 and enhances its activity, driving a transition from a contractile to a synthetic phenotype characterized by increased proliferation, migration, and inflammation—key processes promoting atherosclerosis progression

In this study, we identify the SUMO2/3‐specific protease SENP3 as a critical regulator of VSMC phenotypic switching and demonstrate that its pro-atherogenic effects are mediated through deSUMOylation and stabilization of the transcription factor KLF4. By integrating evidence from cellular models, human atherosclerotic tissues, and genetically modified mice, we establish that the SENP3-KLF4 axis drives VSMCs transition towards a synthetic, pro-inflammatory phenotype and accelerates atherosclerosis development (Fig. 8). These findings reveal a previously unrecognized mechanism underlying vascular plasticity and highlight a therapeutic potential for targeting this axis to decelerate plaque progression and enhance plaque stability.

Our work expands the understanding of SUMOylation in vascular pathobiology. Although prior studies have implicated SENP3 in cancer and metabolic cardiovascular diseases through substrates such as β-catenin, YAP1 and CTH [17, 19, 38, 39], its role in atherosclerosis has not been explored. Building on earlier reports that associated SENP3 with oxidative stress and vascular remodeling [17, 18], we now demonstrate that SENP3 accumulates in human and mouse atherosclerotic lesions and is critically required for PDGF-BB-induced downregulation of contractile markers and acquisition of migratory and proliferative capacities. These results align with the redox-dependent nature of SENP3 [40, 41], and, for the first time, directly link this protease to atherogenic VSMC remodeling.

Notably, smooth muscle-specific deletion of *Senp3* conferred substantial protection against atherosclerosis without major alterations in systemic lipid metabolism, indicating a vessel-autonomous mechanism. In vitro, SENP3 deficiency suppressed migration, proliferation, and inflammatory activation in VSMCs, while preserving contractile gene expression. These findings collectively suggest that SENP3 facilitates atherosclerosis, at least in part, by driving VSMC phenotypic switching and disrupting vascular homeostasis.

A key advance of this study is the identification of KLF4 as a direct substrate of SENP3. KLF4 is a well-established master regulator of VSMC plasticity [42, 43], and its genetic ablation in VSMCs attenuates atherosclerosis and aortic aneurysm formation [27, 29]. Evidence from human genetics further underscores the relevance of KLF4, as genome-wide association studies have implicated it as a susceptibility locus for coronary artery disease [44, 45]. KLF4 is known to be regulated at multiple levels—transcriptionally, e.g., through Sp1 binding following PDGF-BB stimulation [46], and post-transcriptionally via PTMs including phosphorylation, acetylation, and SUMOylation [36, 47]. While SUMO1 conjugation has been shown to modulated KLF4 activity in VSMC proliferation [32] and macrophage polarization [31], the role of SUMO2/3 modifications remained unclear.

Here, we identify lysine 278 (K278) of KLF4 as a key residue modified by SUMO2/3. While SUMO conjugation often promotes proteins for degradation, our data reveal a more nuanced regulatory role for this modification. Specifically, we found that SENP3-mediated deSUMOylation substantially enhances KLF4 protein stability. This conclusion is supported by multiple lines of evidence: SENP3 knockdown accelerates KLF4 turnover, SENP3 overexpression increases KLF4 abundance, and inhibition of the proteasome pathway rescues KLF4 degradation induced by enhanced SUMOylation. Notably, the SUMO-deficient KLF4-K278R mutant exhibited enhanced protein stability and triggered a more pronounced pro-atherogenic phenotype than wild-type KLF4, even without exogenous stimulation. By removing SUMO moieties, SENP3 both stabilizes KLF4 and potentiates its functional output. The specificity of this regulation is underscored by our observation that neither SENP1 nor SENP2 significantly influences KLF4 SUMOylation or stability. Thus, under atherogenic conditions, upregulation of SENP3 represents a critical mechanism that amplifies KLF4 signaling by preventing its degradation and fine-tuning its activity, thereby driving VSMCs plasticity and atherosclerosis progression.

Despite these advances, several limitations remain. While we establish that SENP3-mediated deSUMOylation stabilizes KLF4, it is unclear whether this modification also alters KLF4’s transcriptional specificity or cofactor recruitment. Although our data support a redox-SENP3-KLF4 axis in VSMCs, contributions from other cell types (e.g., macrophages, endothelial cells) cannot be excluded, particularly given the potential off-target activity of *Tagln-Cre*. Moreover, while supporting evidence was obtained in human tissues, most mechanistic studies were conducted in rodent VSMCs; validation in human primary VSMCs would strengthen clinical relevance. We also noted elevated SENP3 expression in adventitial fibroblasts and endothelial cells within human plaques, suggesting roles in inflammation or remodeling beyond VSMCs—a direction worthy of further study. Additionally, this study focused on SUMO2/3 modifications; whether other SUMO paralogs modulate KLF4 remains unknown. Finally, the therapeutic potential of SENP3 inhibition requires further validation in advanced lesion models and non-rodent species to assess efficacy and safety.

In conclusion, this study identifies the SENP3–KLF4 axis as a critical regulator of atherosclerotic plaque progression and instability. SENP3 is upregulated in human and mouse atherosclerotic lesions, where it deSUMOylates and stabilizes KLF4, driving VSMCs phenotypic switching, inflammation, and matrix remodeling. Genetic disruption of SENP3 or impairment of KLF4 SUMOylation attenuates these pro-atherogenic effects. Our results highlight SENP3-mediated deSUMOylation of KLF4 as a promising therapeutic target for stabilizing vulnerable plaques.

## Materials and methods

### Human specimens

Human arterial specimens were collected from patients undergoing limb amputation after obtaining written informed consent. Atherosclerotic popliteal arteries were utilized for this study. The study cohort included 3 male patients with a mean age of 68 ± 8 years, all of whom had hypertension. Additional comorbidities included diabetes mellitus (2/3) and dyslipidemia (2/3). All procedures were approved by Ethics Committee of Renji Hospital, Shanghai Jiao Tong University School of Medicine, and were conducted in compliance with the Declaration of Helsinki. Tissues were processed using standard methods, including formalin fixation, paraffin embedding, and sectioning at 8 μm thickness for histological and immunofluorescence analyses.

### Animals

*ApoE*^−/−^ (Stock No. 002052) and *Tagln-Cre* (Stock No. 017491) transgenic mice *on a* C57BL/6 background were sourced from Jackson Laboratory. *Senp3*^*flox/flox*^ mice were generously provided by Prof. Jing Yi (Shanghai Jiao Tong University). Experimental *ApoE*^−/−^;*Senp3*^*flox/flox*^;*Tagln-Cre* mice were generated through standard crossbreeding strategies. All animals were housed under specific pathogen-free (SPF) conditions at 22 °C with a 12-h light/dark cycle and had free access to food and water. The animal study protocol was approved by the Institutional Animal Care and Use Committee at Shanghai Chest Hospital (Permit No. KS23042).

### Atherosclerosis model

Atherosclerosis was induced in 8-week-old mice via partial ligation of the left common carotid artery (LCCA) and the left renal artery (LRA) as described previously [33, 34]. Mice were fed a Western diet (Research Diets, D12079B) for 4 weeks following surgery.

### Magnetic resonance imaging

In vivo plaque imaging was performed using a 7.0 T small-animal MRI system (Siemens Medical Inc., Erlangen, Germany) with a dedicated mouse RF coil. Under anesthetized (1.5% isoflurane in 2.5 L/min O_2_), physiological parameters (respiration, body temperature, and heart rate (ECG)) were continuously monitored. T2-weighted RARE sequences with fat suppression were acquired under respiratory and ECG triggering using the following parameters: repetition time (TR) = 2200 ms, echo time (TE) = 12 ms, field of view = 25.0 mm × 25.0 mm, matrix = 256 × 256, slice thickness = 1.0 mm, and slice gap = 0.6 mm. Total imaging time was approximately 1 h per mouse.

### Histology and Immunofluorescence

After euthanasia, heart and vascular tissues were harvested, perfused with PBS, and fixed. Tissue samples were embedded in either optimal cutting temperature (OCT) compound or paraffin and sectioned at a thickness of 5 µm. For histological evaluation, sections were subjected to hematoxylin and eosin (H&E), Masson’s trichrome, and Oil Red O staining. For *en face* lesion analysis, aortas were dissected, longitudinally opened, and stained with Oil Red O to quantify lipid-rich plaque areas.

Immunofluorescence staining was performed on fixed sections using primary antibodies against α-SMA (1:100, Abcam), SENP3 (1:100, CST), and KLF4 (1:100, Abcam), followed by appropriate fluorophore-conjugated secondary antibodies. Images were acquired using Leica DM2500 (Leica Microsystems, Germany) or Zeiss LSM710 confocal microscope (Carl Zeiss, USA) and analyzed with ImageJ software.

### Serum lipid profiling

Blood was collected from the retro-orbital sinus. Serum lipid levels were measured enzymatically using Infinity™ reagents (Thermo DMA, USA) according to the manufacturer’s instructions.

### Cell culture and treatments

Primary VSMCs were isolated from the thoracic aorta of mouse or rat using established protocols. Cells were maintained in high glucose Dulbecco's Modified Eagle's Medium (DMEM; Thermo Fisher Scientific) supplemented with 10% fetal bovine serum (FBS) at 37 °C under 5% CO₂. VSMCs between passages 3 and 8 were utilized in experiments to ensure experimental reproducibility. To induce phenotype switching, cells were treated with platelet-derived growth factor-BB (PDGF-BB, 40 ng/ml; Sigma-Aldrich), oxidized low-density lipoprotein (oxLDL, 20 μg/ml; Sigma-Aldrich) or cholesterol crystals (CC, 1 mg/ml; Sigma-Aldrich).

### Plasmids and transfection

The full-length human KLF4 cDNA was subcloned into the pcDNA3.1 vector to generate the Flag-tagged KLF4 expression plasmid. Plasmids for GFP-SENP3, its catalytically inactive mutant (GFP-SENP3/C532A), and HA-tagged SUMO1, SUMO2, and SUMO3 plasmids were employed as previously described [17, 19]. Using the wild-type Flag-KLF4 as a template and the QuikChange mutagenesis kit (Stratagene, La Jolla, CA, USA), the Flag-KLF4-K278R mutant was created via site-directed mutagenesis. Subsequent transfection of HEK293T cells with the relevant plasmids were performed using Lipofectamine 8000 (Beyotime Biotechnology, Nanjing, China), according to the manufacturer’s protocol.

### Lentiviral transduction

Lentiviral vector encoding shRNA targeting SENP3 or KLF4 were cloned into the PGMLV-hU6-MCS-CMV-ZsGreen1-PGK-Puro-WPRE plasmid (Genomeditech, Shanghai, China). The shRNA sequences used were as follows: SENP3: 5’-ggtactacagctgatccaatc-3’, 5’-ggctcaatgaccaggtgatga-3’, or 5’-gcatattgccaagtatctaca-3’; KLF4: 5’-ggacctagactttatcctttc-3’, 5’-gctcctctacagccgagaatc-3’, or 5’-ggtcatcagtgttagcaaagg-3’. Lentiviral particles were produced by co-transfecting HEK293T cells with the respective shRNA vectors and packaging plasmids. The viral supernatant was collected 48 h post-transfection, concentrated, and resuspended in PBS. VSMCs were infected with the viral particles at a multiplicity of infection (MOI) of 30.

### Migration and proliferation assays

Cell migration capacity was evaluated using both Transwell and scratch wound assays. In the Transwell system, 2 × 10^4^ cells were plated in serum-free medium into the upper chamber (8.0 μm pore; Corning, NY, USA), while the lower chamber contained PDGF-BB (40 ng/ml) as a chemoattractant. Following a 24-h incubation, cells that had traversed the membrane were fixed, stained with 0.5% crystal violet, and quantified by manually counting. For the scratch wound assay, a uniform wound was introduced into confluent monolayers using a pipette tip, followed by PDGF-BB (40 ng/mL) treatment. Wound closure was documented at 0 and 24 h under microscope, and migration was quantified by calculating the healed area with ImageJ software.

Cell proliferation was evaluated using the BeyoClick™ EdU-488 Kit (Beyotime Biotechnology, Nanjing, China). Following treatment with PDGF-BB (40 ng/mL), cells were incubated with 10 μM EdU for 2 h, fixed, and processed according to the manufacturer’s instructions. The percentage of EdU-positive cells was quantified using a Cytation 3 imaging system (BioTek Instruments Inc., Winooski, VT, USA).

### Quantitative PCR (qPCR)

Total RNA was isolated using TRIzol reagent (Invitrogen, Eugene, OR, USA). Complementary cDNA (cDNA) synthesis was carried out with the PrimeScript® RT Reagent Kit (Takara, Japan). Quantitative real-time PCR assays were performed on a Roche 7300 System (Roche Applied Science) with the TB Green® Premix Ex Taq™ II (Tli RNaseH Plus) Kit (Takara Bio). Relative gene expression was determined using GAPDH as an internal control and calculated via the 2^ − ΔΔCt method. Corresponding primer sequences are listed in Table S1.

### Immunoblotting and immunoprecipitation

Cells were lysed in an ice-cold buffer composed of 1% Triton X-100, 150 mM NaCl, 10 mM Tris–HCl (pH 7.4), 1 mM EDTA, 1 mM EGTA (pH 8.0), 0.2 mM sodium orthovanadate, 1 mM PMSF, and protease inhibitors. The resulting lysates were subjected to sonication, followed by centrifugation at 12,000 rpm for 15 min at 4 °C. Protein concentration was quantified using a BCA assay Kit (Thermo Fisher Scientific, USA). Subsequently, proteins were resolved by SDS–PAGE and electrophoretically transferred to polyvinylidene fluoride (PVDF) membranes (Amersham). After blocking with 5% non-fat milk in TBST for 1 h at 37 °C, the membranes were probed with specific primary antibodies at 4℃ overnight. The following antibodies were used: SENP3 (CST, 1:1000), SM22α (CST, 1:1000),α-SMA (Abcam, 1:1000), KLF4 (Abcam, 1:1000), SUMO1(CST, 1:1000), SUMO2/3 (CST, 1:1000), Flag (CST, 1:1000), GFP (CST, 1:1000), RGS (Beyotime, 1:1000), HA (CST, 1:1000), MMP2 (ABclonal, 1:1000), MMP9 (ABclonal, 1:1000), Col1a1 (ABclonal, 1:1000), β-actin (Abclonal, 1:1000), and GAPDH (CST, 1:1000). After washing, membranes were incubated with horseradish peroxidase (HRP)-conjugated secondary antibodies for 1 h at 37℃. Protein signals were finally visualized using chemiluminescence (ECL) reagents (Thermo Fisher Scientific, Waltham, MA, USA).

For co-immunoprecipitation assays, cells were harvested and lysed in Pierce IP Lysis buffer (Thermo Fisher Scientific, Cat# 87,788) supplemented with a protease inhibitor cocktail (Roche, Cat# 11,697,498,001) and 20 mM N-ethylmaleimide (Sigma-Aldrich, Cat# E3876). The clarified lysates were then subjected to incubate with either anti-KLF4 or anti-Flag magnetic beads (Sigma-Aldrich, Cat# M8823) overnight at 4 °C. Following a series of washes, the immunoprecipitated proteins were eluted from the beads and prepared for subsequent immunoblotting analysis.

### Statistical analysis

Statistical data are presented as the mean ± standard error of the mean (SEM). Intergroup comparisons were evaluated for statistical significance using either Student’s t-test or one-way ANOVA. A p-value < 0.05 was considered statistically significant. Specific statistical tests and results are detailed in the respective figure legends. All statistical analyses and graphical representations were performed with GraphPad Prism software (version 9).

## Supplementary Information

Supplementary Material 1.

## Data Availability

The datasets generated and/or analyzed during the current study are not publicly available due to ethical restrictions and patient confidentiality agreements, as well as ongoing data analysis. However, the data may be available from the corresponding author upon reasonable request.
