# Supplementary material for: Sentrin‐specific protease 3 (SENP3)-mediated Krüppel-like factor 4 (KLF4) deSUMOylation regulates vascular smooth muscle cell phenotypic switching in atherosclerosis
Source: Mol Biomed. 2025 Nov 27;6:124. doi: 10.1186/s43556-025-00365-5 (PMC12660617; doi:10.1186/s43556-025-00365-5)
Supplement: Supplementary file 1 — Supplementary Material 1. [file 43556_2025_365_MOESM1_ESM.docx]

**Supplemental data**

**Sentrin‐specific proteases 3 (SENP3)-mediated Krüppel-like factor 4 (KLF4) deSUMOylation Regulates Vascular Smooth Muscle Cell Phenotypic Switching in Atherosclerosis**

Zi Wang^1, 2 a^, Yinan Wang^3 a^, Ruosen Yuan^4^, Qingqi Ji^2^, Yanjie Li^2^, Huanhuan Huo^2^, Guo Zhou^2^, Xiangming Yan^2^, Linghong Shen^2^, Zhaohua Cai^2, *^, Ben He^2, *^

^1^ Department of Cardiology, Ren Ji Hospital, Shanghai Jiao Tong University School of Medicine, Shanghai, China, 200030

^2^ Department of Cardiology, Shanghai Chest Hospital, Shanghai Jiao Tong University School of Medicine, Shanghai, China, 200030

^3^ State Key Laboratory of Systems Medicine for Cancer, Renji-Med X Clinical Stem Cell Research Center, Ren Ji Hospital, Shanghai Jiao Tong University School of Medicine, Shanghai, China, 200127

^4^ Department of Cardiology, Rui Jin Hospital, Shanghai Jiao Tong University School of Medicine, Shanghai, China, 200025

^a^ The co-first authors contribute equally to the article.

^*^ Corresponding authors:

Ben He, M.D., Ph.D., Department of Cardiology, Shanghai Chest Hospital, Shanghai Jiao Tong University School of Medicine, Shanghai, China, 200030; E-mail: heben2410@126.com.

Zhaohua Cai, M.D., Ph.D., Department of Cardiology, Shanghai Chest Hospital, Shanghai Jiao Tong University School of Medicine, Shanghai, China, 200030; E-mail: zcai5@yahoo.com.

ORCID iD: Zi Wang: 0000-0002-1727-8397

**
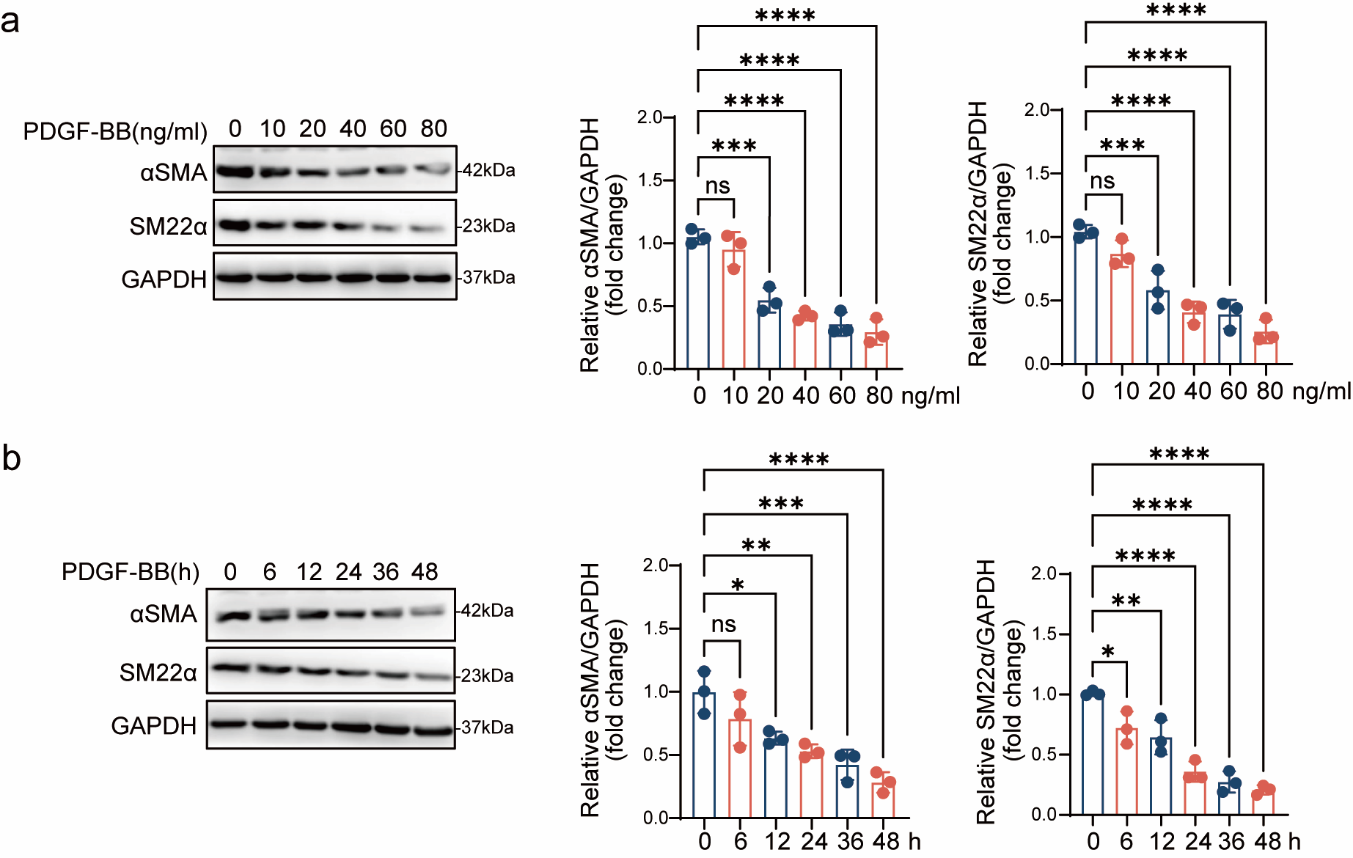
**

**Fig. S1. Optimization of PDGF-BB treatment for inducing phenotypic switching in primary rat VSMCs**

**a**. Dose response analysis of contractile marker proteins (αSMA and SM22α) following 24 h PDGF-BB treatment.

**b**. Time dependent changes in αSMA and SM22α expression upon stimulation with 40 ng/mL PDGF-BB.

Based on these results, treatment with 40 ng/mL PDGF-BB for 24 h was selected for all subsequent experiments to effectively induce the synthetic phenotype of VSMCs.


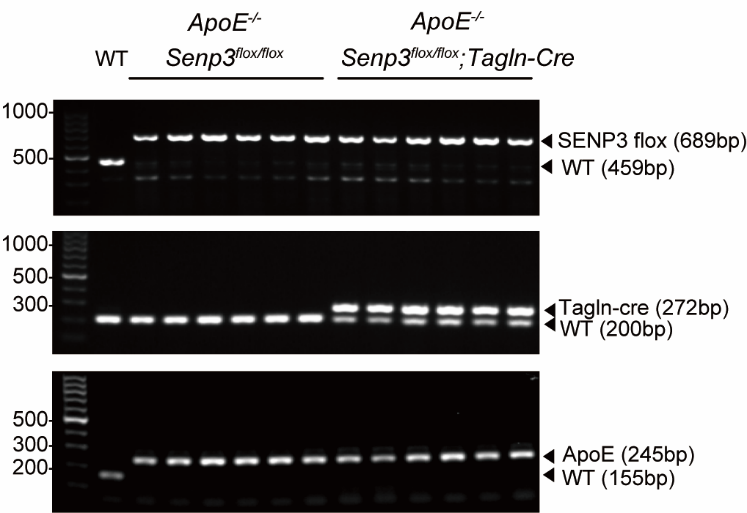


**Fig. S2.** **Generation of ApoE^−/−^;Senp3*^flox/flox^*;*Tagln-Cre* mice**

Genotype analysis of wild-type (WT), ApoE^−/−^;Senp3*^flox/flox^*, and ApoE^−/−^;Senp3*^flox/flox^*;*Tagln-Cre* mice.

**Supplementary Table 1. The primer sequences for qPCR in this study**

| Primer | Primer sequence (5’ to 3’) |
| --- | --- |
| SENP3 | Forward 5’- GCACCTCGCTGACATTCCACTG - 3’ |
|  | Reverse 5’- ATCCTCTGCCATGAGAGCACCTAG - 3’ |
| KLF4 | Forward 5’- TTCAACCTGGCGGACATCAATGAC - 3’ |
|  | Reverse 5’- GAGGCTGCTGTGGCGGAATG - 3’ |
| GAPDH | Forward 5’- GACATGCCGCCTGGAGAAAC - 3’ |
|  | Reverse 5’- AGCCCAGGATGCCCTTTAGT - 3’ |
| Acta2 | Forward 5’- GCCACTGCTGCTTCCTCTTCTTC - 3’ |
|  | Reverse 5’- TGCCCGCCGACTCCATTCC - 3’ |
| Tagln | Forward 5’- GTGACCAAGAACGATGGACACTACC - 3’ |
|  | Reverse 5’- GACTGTCTGTGAACTCCCTCTTATGC - 3’ |
| Myh11 | Forward 5’- CGCCAGAACAAGGAACTCCGAAG - 3’ |
|  | Reverse 5’- GCCTCTAGTGCCGCAACAGTG - 3’ |
| Itgb1 | Forward 5’- TGCCAACCAAGTGACATAGAGAATCC - 3’ |
|  | Reverse 5’- TGCCATCCCTTTGCTGCGATTG - 3’ |
| TNF-α | Forward 5’- ATGGGCTCCCTCTCATCAGTTCC - 3’ |
|  | Reverse 5’- GCTCCTCCGCTTGGTGGTTTG - 3’ |
| IL-6 | Forward 5’- ACTTCCAGCCAGTTGCCTTCTTG - 3’ |
|  | Reverse 5’- TGGTCTGTTGTGGGTGGTATCCTC - 3’ |
| IL-18 | Forward 5’- CGACCGAACAGCCAACGAATCC - 3’ |
|  | Reverse 5’- TCACAGATAGGGTCACAGCCAGTC - 3’ |
| IL-1β | Forward 5’- CTCACAGCAGCATCTCGACAAGAG - 3’ |
|  | Reverse 5’- TCCACGGGCAAGACATAGGTAGC - 3’ |
| TGF-β | Forward 5’- GACCGCAACAACGCAATCTATGAC - 3’ |
|  | Reverse 5’- CTGGCACTGCTTCCCGAATGTC - 3’ |
